# 16 Spectrum of infections during juvenile idiopathic arthritis

**DOI:** 10.1093/rheumatology/keac496.012

**Published:** 2022-10-07

**Authors:** Nouha Dissem, Safa Rahmouni, Soumaya Boussaid, Houssem Tbini, Samia Jemmali, Sonia Rekik, Khaoula Zouaoui, Hela Sahli, Mohammed Elleuch

**Affiliations:** 1 Department of Rheumatology, La Rabta Hospital, Tunis, Tunisia; 2 University of Tunis El Manar Department of Rheumatology, La Rabta Hospital, Tunis, Tunisia

## Abstract

**Background:**

The incidence of infections in patients with chronic inflammatory rheumatic disease is increased. It is often due to the disease itself and to the immunosuppressive treatments used.

**Objectives:**

To assess the incidence of infections during JIA.

**Methods:**

We conducted a repeated cross-sectional study including 29 patients followed for JIA according to the International League of Associations for Rheumatology (ILAR) criteria over a period from 1994 to 2022. Sociodemographic and anthropometric parameters, clinical data, biological assessments, and prescribed therapies were collected. We identified patients who had at least one infectious episode during their follow-up.

**Results:**

There were 17 women and 12 men. The mean age was 35.69 ± 11.72 [18–61] years. The polyarticular form was seen in 55.2% of cases. The mean age of disease onset was 11.10 ± 4.25 [2–16] years. The average disease duration was 24.48 ± 12.76 [1–47] years.

Diabetes and arterial hypertension were the main comorbidities associated with JIA, observed in 13.8% of cases each. At least one extra-articular manifestation was noted in 16 cases: pulmonary (3 cases), cardiac (4 cases), renal (2 cases), cutaneous (4 cases) and ocular (7 cases).

The most prescribed DMARDs was Methotrexate in 79.3% (*n* = 23), biotherapy was used in 3 (10.3%), NSAIDs and corticosteroids were used in 62.1% (*n* = 18) and 69% (*n* = 20) respectively.

All the infections observed in our population were of community origin. Urinary tract infection was the most common infection (*n* = 5). Bronchopulmonary infections were observed in 2 cases including a case of tuberculosis. Sub periosteal abscess of the femur was also seen in one of the patients.

Regarding the SARS-CoV-2 infection, 6 patients were infected, 2 of whom required hospitalization, including one in the intensive care.

**Conclusion:**

The risk of infections is increased during JIA. This is due to the immunosuppression induced by the disease, the treatment, and comorbidities.

